# Synergies of Quorum Sensing and Biofilm Dynamics in the Bioremediation of Emerging Medical Organic Pollutants

**DOI:** 10.1155/tswj/5568616

**Published:** 2026-04-28

**Authors:** Yohannes Tsegay Teklay

**Affiliations:** ^1^ Faculty of Biotechnology, Mekelle Institute of Technology, Mekelle University, Mekele, Ethiopia, mu.edu.et

**Keywords:** biofilm, bioreactor, emerging organic pollutants, quorum sensing, wastewater

## Abstract

Emerging organic pollutants in medical waste present significant environmental challenges. Bioremediation is an eco‐friendly and cost‐effective solution, leveraging natural processes to effectively mitigate these risks. So, this review aims to discuss the role of microbial biofilm and quorum sensing in the bioremediation of these pollutants, with a special focus on their mechanism of action, application, and potential. The review begins with an overview of emerging organic pollutants, the importance of bioremediation, the basics of quorum sensing, and its significance in a microbial consortium. Key findings indicate that technological applications such as engineered biofilm bioreactors, electroactive biofilms in microbial fuel cells, co‐culture systems, and genetic engineering of QS pathways significantly accelerate pollutant mitigation compared to traditional methods. For instance, specific case studies (e.g., *Pseudomonas aeruginosa* in pharmaceutical degradation) demonstrate the efficacy of QS‐mediated metabolic control. A key conclusion is that leveraging these integrated QS‐biofilm systems can surpass conventional waste degradation approaches. However, limitations include the difficulty of scaling up laboratory nanobioremediation systems and the complexity of interspecies signaling in real‐world applications. Future research bottlenecks must prioritize investigating the stability of QS signals within complex wastewater matrices impacted by variables like pH and indigenous quorum‐quenching microorganisms and developing precise biofilm control strategies through QS manipulation to optimize architecture for targeted degradation. Bridging these gaps through real‐scale validation is essential to transition these promising laboratory‐scale technologies into practical environmental applications. This review serves as a benchmark for developing immediate, bio‐based solutions to mitigate the risks posed by EMOPs.

## 1. Introduction

Medical waste, or healthcare waste, is waste released from several areas, such as hospitals, laboratories, clinics, and research areas. These can be classified into several types, such as chemical waste [1], pharmaceutical waste [2], radioactive waste [3], pathological waste [4], sharps, and infectious waste [5]. When these hazardous substances are released, they cause environmental damage [6]. For instance, the open burning and low‐temperature incineration of these wastes can lead to the release of dioxins, furans, and particulate matter, which then lead to an impact on the environment and human health [7]. Moreover, water sources contaminated with these can harm aquatic life and then enter the human food chain, posing a health risk [8].

Likewise, emerging medical organic pollutants are a group of emerging organic pollutants (EOPs) that originated from medical activities, including chemicals used in medical treatments, personal care products, and pharmaceuticals [9, 10]. Pharmaceutical and personal care products from human or veterinary medicine enter the environment through improper disposal, wastewater, and agricultural runoff. Then they can persist and accumulate, leading to disrupting the ecosystem [10, 11]. Pharmaceuticals, especially antibiotics, contaminate the water system globally and pose a risk to human health. Due to incomplete wastewater treatment, these pollutants cause accumulation, altering microbial communities and leading to antibiotic resistance. So, wastewater treatment is the major source of this contamination [12, 13]. Moreover, personal care products, such as preservatives, fragrances, and disinfectants, can persist for a long time in the environment and risk aquatic life and human health [14, 15].

Therefore, improper healthcare waste worsens the environmental contamination of water and soil [16, 17], as traditional treatment methods are ineffective in completely degrading [18].

Medical EOPs pose unique challenges to conventional biodegradation due to their high biological activity at trace concentrations, inherent antimicrobial properties, and often complex, recalcitrant structures that bypass standard metabolic induction in planktonic cells [19]. Unlike traditional planktonic systems, where individual cells are vulnerable to the toxic shocks of pharmaceuticals, the synergy between quorum sensing (QS) and biofilm architecture provides a superior remedial mechanism [20, 21]. QS functions as a sophisticated regulatory “command center,” synchronizing the expression of catabolic genes and the secretion of specialized enzymes in response to localized cell density [22]. Simultaneously, the biofilm’s extracellular polymeric substance (EPS) matrix serves as a protective barrier and an adsorbent that traps trace‐level medical EOPs, increasing their bioavailability and protecting the microbial community from potential growth‐inhibiting effects [23]. This collaborative behavior enables efficient mineralization of complex medical compounds that would otherwise remain untreated by conventional biological processes.

### 1.1. Mechanisms of QS in Biofilm Development

A biofilm is a structured community of microbial cells irreversibly attached to a surface and encapsulated within a self‐produced matrix of EPS, including polysaccharides, proteins, and eDNA. Biofilm formation is a dynamic, multistage process involving initial reversible attachment of planktonic cells, irreversible adhesion, microcolony formation, maturation into a complex 3D structure with water channels, and eventual dispersal [24, 25]. QS is the critical cell‐to‐cell communication system that tightly regulates this cycle in a population density‐dependent manner through the production and detection of small signaling molecules called autoinducers (AIs) [26]. In Gram‐negative bacteria, for example, N‐acyl homoserine lactones (AHLs) act as AIs, which, upon reaching a threshold concentration, bind to intracellular receptors to trigger the collective expression of target genes [27]. This gene regulation is not merely for structural development; it is a key physiological switch. Specifically, for EOP degradation, QS activation within mature biofilms orchestrates a coordinated metabolic response, enhancing the secretion of specialized extracellular enzymes that break down complex and often toxic EOPs [28]. The dense, protective EPS matrix, whose synthesis is significantly enhanced by QS signals, shields the embedded microbes from the inherent toxicity of medical pollutants and concentrates these trace‐level contaminants, thereby facilitating efficient, community‐wide bioremediation that is unattainable by individual, vulnerable planktonic cells [29].

### 1.2. Rationale for Integrating Biofilms and QS in EOP Bioremediation

Biofilm‐mediated remediation is an effective approach for the treatment of environmental waste, offering advantages over traditional methods due to its efficiency and cost‐effectiveness. Microbial communities adhered to surfaces, providing a protective environment for cells and enhancing their ability to degrade toxic pollutants [30]. Moreover, QS plays a role in regulating biofilm formation and dispersal. Besides, QS signals can be engineered to control biofilm communities for the remediation of pollutants, improving degradation kinetics, and enhancing the overall process [31]. The matrix of biofilm facilitates the exchange of genetic material, metabolites, and signal molecules between cells, as well as the sorption and immobilization of pollutants [32]. These properties make biofilm‐mediated remediation a powerful tool for addressing various toxic organic pollutants in the environment [30]. The specific contributions of both biofilms and QS to this process are summarized in Table 1.

**TABLE 1:** Rationale for integrating biofilms and quorum sensing in emerging organic pollutant bioremediation.

Feature	Biofilm contribution	Quorum sensing (QS) contribution	Integrated rationale	References
Microbial protection and survival	Provides a protective, self‐synthesized extracellular polymeric substance (EPS) matrix that shields microbes from environmental stressors (e.g., pH changes, UV radiation, high pollutant concentrations, predation, and desiccation).	Regulates genes for stress tolerance and coordinates collective defense mechanisms, improving the community’s overall survival rate in toxic environments.	Enhanced survival and persistence of the microbial community in harsh, contaminated conditions where free‐floating (planktonic) cells would perish.	[33, 34]
Enhanced pollutant access and degradation	The EPS matrix acts as a natural biosorbent, binding and concentrating hydrophobic organic pollutants, thereby increasing their local concentration and bioavailability to the embedded microbes.	QS regulates the production of biosurfactants and extracellular enzymes, which further solubilize and break down complex, often insoluble, organic compounds, making them more accessible for microbial metabolism.	Increased efficiency of pollutant degradation through a combined mechanism of initial binding/concentration (biofilm matrix) and increased bioavailability/enzymatic breakdown (QS‐regulated processes).	[35–37]
Metabolic diversity and efficiency	The three‐dimensional structure of the biofilm creates diverse microniches with varying conditions (e.g., oxygen gradients), allowing both aerobic and anaerobic microorganisms to coexist and perform a variety of metabolic processes simultaneously.	Facilitates cell‐to‐cell communication and horizontal gene transfer (HGT), enabling a diverse microbial consortium to coordinate complex catabolic pathways for the degradation of a wide range of organic pollutants.	Optimized and often faster degradation of complex pollutant mixtures that would be difficult for a single species or planktonic cells to break down alone.	[31, 38, 39]
Process control and optimization	Forms stable, high‐density biomass ideal for engineered systems like bioreactors, enabling high efficiency and retention of active microbial populations over long periods.	QS pathways can be manipulated (e.g., using QS enhancers or inhibitors) to control biofilm formation, structure, and activity, allowing engineers to optimize bioremediation performance for specific pollutants.	The ability to engineer and manage effective and stable bioremediation systems with controllable, predictable degradation kinetics.	[40, 41]

Therefore, biofilm‐mediated remediation is not merely an alternative but a structurally complex strategy for mitigating environmental pollutants [42]. The integration of QS into these systems provides a regulatory layer that converts passive microbial adhesion into an active, density‐dependent degradative engine. Furthermore, QS regulation can increase biomass yields under substrate toxicity stress, providing a more robust biological shield against harsh medical pollutants. A critical analysis of the synergistic advantages and inherent limitations of these integrated systems is presented in Table 2.

**TABLE 2:** Critical analysis of integrated biofilm and quorum‐sensing systems.

Feature	Synergistic advantage	Critical trade‐offs and limitations	Evidence (microcosm/stats)	References
Pollutant access	QS regulates **biosurfactants**, solubilizing insoluble pollutants.	**Mass-transfer bottlenecks**: EPS reduces diffusivity by up to **80%**.	Microcosm: *P. aeruginosa* shows increased PAHs degradation via QS‐regulated surfactants.	[43]
Metabolic control	QS triggers specific enzymatic pathways (e.g., catabolic genes).	**Signal instability**: pH and temperature changes in field sites disrupt signal molecules.	Statistical: Exogenous AHLs increased heavy metal removal by **∼37%** in wastewater.	[44]
Structural stability	Biofilms protect cells; QS manages maturation and prevents premature dispersal.	**Biofouling/detachment**: QS activation can lead to excessive growth, causing reactor clogging.	Study: QQ beads reduced fouling by **50%** in membrane reactors.	[45, 46]
Resource efficiency	Limits gene expression to high cell densities, saving metabolic energy.	**Metabolic load**: Engineered QS circuits can reduce the growth rate of the carrier strain.	Microcosm: *E. coli* biofilm formation vs. N/P concentration ratios.	[47]

## 2. Synergies of QS and Biofilm Dynamics in Bioremediation of Emerging Medical Organic Pollutants (EMOPs)

### 2.1. Mechanistic Pathways of QS‐Biofilm Synergy in Pollutant Degradation

#### 2.1.1. Biological Mechanism

The bioremediation of EMOPs is understood to rely on a sophisticated causal synergy where QS acts as the operational “command” for the metabolic “machinery” of the biofilm. Under this synergistic framework, the accumulation of signaling molecules like AHLs triggers the synchronized transcription of specific catabolic enzymes, such as laccases and heme‐peroxidases, which are essential for degrading recalcitrant pharmaceuticals [48]. Simultaneously, QS‐mediated community responses provide the biofilm with heightened resilience against “toxic shock” by upregulating stress‐defense genes, efflux pumps, and EPS production, creating a stabilized environment where degradation can occur despite the high toxicity of medical waste [49]. This process is further optimized in mixed‐culture consortia through the role of universal signals like Autoinducer‐2 (AI‐2), which coordinate interspecies co‐metabolism and cross‐feeding, ensuring that complex pollutant cocktails are completely detoxified through the collaborative efforts of a stable microbial community [31].

The bioremediation of EMOPs is increasingly defined by the synergy between QS and biofilm dynamics, where signaling molecules like AHLs and AI‐2 synchronize microbial metabolic pathways to enhance the degradation of complex pharmaceutical wastes [43]. While the biofilm matrix provides the structural resilience necessary to shield microbes from the toxic shock of antibiotics [50], QS acts as the regulatory trigger for the expression of specific catabolic enzymes and the optimization of the EPS architecture [51]. Despite these laboratory‐scale successes, a critical challenge remains in the environmental stability of QS signals, which are often susceptible to degradation by fluctuating pH, temperature, and indigenous quorum‐quenching microorganisms within real‐world healthcare wastewater [52].

For instance, certain strains of *Labrys portucalensis F11* can degrade pharmaceutical waste such as fluoxetine [53]. In this case, QS‐mediated biofilm formation allows more effective degradation of fluoxetine, thanks to improved nutrient transfer and protection from environmental stress [54]. Another example is bacteria that degrade estrogenic compounds. As estrogen concentration increases, these bacteria communicate via signals that promote biofilm development. The biofilm acts as a matrix that supports the bacterial community and then allows the degradation of estrogen [55]. Quantitative data reflecting the enhanced degradation efficiency of various EMOPs through these integrated systems are detailed in Table 3.

**TABLE 3:** Quantitative data: synergistic degradation of emerging medical organic pollutants.

Medical organic pollutant	Class	Quantitative metric	QS–biofilm synergistic role	References
Sulfamethoxazole	Antibiotic	90.8% removal efficiency in electroactive biofilms.	QS synchronizes the expression of degradation genes (*sadABC*); electroactive biofilms provide enhanced electron donors for metabolic breakdown.	[56]
Diclofenac	NSAID (painkiller)	97.8% degradation within 6 days by optimized bacterial strains.	QS regulates biofilm structure; synergistic treatment with co‐substrates reduces toxic intermediate accumulation.	[57]
Ciprofloxacin	Antibiotic	Log‐scale reduction in biomass recovery following signal loss.	Biofilm EPS matrix (QS‐regulated) provides up to a 1000‐fold increase in resistance, allowing for sustained degradation under toxic shock.	[58]
Ibuprofen	NSAID (nonsteroidal anti‐inflammatory drug)	Above 70% removal efficiency.	Quorum sensing (QS) inhibition reduces biofilm formation and virulence, aiding overall microbial degradation.	[59]

##### 2.1.1.1. Activation of Specific Enzymes

QS‐biofilm synergies serve as the metabolic switch that synchronizes the production of degradative enzymes when a critical bacterial cell density is reached [60]. In bacterial systems, this is primarily mediated by AHL signaling molecules, which trigger the expression of specific catabolic genes [61]. In the presence of emerging medical pollutants such as pharmaceuticals and personal care products, QS triggers the expression of specific enzymes for the degradation of these compounds [62, 63]. Enzymes such as hydrolases, oxidoreductases, and transferases play a crucial role in enhancing the degradation rate of complex pharmaceutical pollutants [64–67]. So, these enzymes make QS‐biofilm the best mechanism for the degradation of the toxic medical pollutants.

While bacterial QS relies on AHLs, fungi utilize distinct signaling molecules, such as farnesol and tyrosol, to regulate their physiological responses. To maintain conceptual clarity, it is noted that fungi, particularly white‐rot fungi (e.g., *Trametes versicolor*), contribute to EOP degradation through a distinct, nonspecific enzymatic system [68]. Extracellular enzymes such as laccase and manganese peroxidase have been shown to degrade over 90% of diclofenac and sulfamethoxazole within 48 h by generating highly reactive radicals that attack the aromatic rings of these pharmaceuticals [69]. Furthermore, fungal cytochrome P450 monooxygenases catalyze the hydroxylation of complex compounds such as carbamazepine (CBZ), a drug notoriously recalcitrant to bacterial degradation [70]. This indicates that synergies of QS‐biofilm–regulated enzymes are important for the degradation of EOPs.

##### 2.1.1.2. Synergistic Interactions Among Microbial Communities

Due to their complex structures and potential toxicity of medical EOPs, such as personal care products and pharmaceuticals, they pose significant challenges for microbial degradation [71]. However, bacteria utilize QS–biofilm mechanisms to enhance their collective degradation capabilities [72]. In microbial communities, QS of biofilm facilitates communication that allows bacteria to coordinate their action based on population density. This coordination is crucial during the degradation of complex organic pollutants [60]. For instance, one species may produce certain enzymes that initiate the breakdown of pollutants, while another species can utilize the resulting metabolites, thereby enhancing the overall degradation process [73]. This synergistic cooperation is evident in QS‐biofilm–forming communities, where QS regulates the formation of biofilm that provides a protective environment for microbial interaction and degradation [30, 74].

#### 2.1.2. Technological Innovations

##### 2.1.2.1. Biofilm Bioreactors Enhanced by QS Control

Recent research indicates that directly applying QS regulation can enhance the operational efficiency of moving bed biofilm reactors (MBBR) and membrane biofilm reactors (MBR) in biological wastewater treatment [75, 76]. Biofilm bioreactors outperform suspended growth systems in treating healthcare wastewater by maintaining a high biomass age (sludge retention time), which is critical for the slow‐growing nitrifiers that co‐metabolically degrade medical EOPs [77, 78].

There are several types of biofilm bioreactors, such as fixed‐bed reactors, membrane bioreactors, and trickling filters [79, 80]. As discussed below:

In the degradation of EMOPs, the operational performance of advanced bioreactors, such as MBBRs [81] and MBRs [82], is significantly enhanced through strategic QS regulation. Research has demonstrated that the direct addition of exogenous signaling molecules, primarily AHLs like C6‐HSL and C8‐HSL, can accelerate biofilm start‐up and increase biomass adhesion to carriers. This chemical signaling fosters a causal synergy where the structural biofilm matrix acts as a protective shield, allowing the community to maintain metabolic activity even when exposed to the high toxicity of antibiotics. These “signal‐guided” systems are particularly effective at improving reactor stability against environmental stressors, such as toxic shocks from high‐concentration medical waste or extreme temperature fluctuations [83]. Thus, by strategically modulating QS‐mediated signaling, these bioreactors cultivate a synchronized microbial architecture that optimizes the secretion of specialized degradative enzymes targeting complex pharmaceutical compounds.

Moreover, a study by Zhao et al. [78] demonstrated that MBRs can help to treat pharmaceutical waste from hospital wastewater. The mechanism of remediation is driven by “counter‐diffusion,” where oxygen supplied through gas‐permeable membranes meets EOPs diffusing from the bulk liquid. This allows for the simultaneous removal of antibiotics and nitrogenous compounds within a single biofilm structure [84]. Moreover, the MBBR is also important for the degradation of hospital wastewater containing EOPs, utilizing plastic carriers for biofilm growth [81]. Quantitative performance in MBBRs is highly sensitive to the hydraulic retention time (HRT) and carrier filling ratio [85]. For example, a study conducted on this system at Al‐Batool Teaching Hospital in Iraq demonstrated that the removal efficiency of BOD, COD, and TSS was 79.5%, 74.5%, and 78%, respectively, by optimizing the contact time between the biofilm and hospital influent [86]. Mechanistically, MBBRs enhance EOP degradation because the continuous movement of carriers reduces the stagnant liquid boundary layer, increasing the mass transfer flux of micropollutants into the biofilm matrix [87]. For highly recalcitrant pharmaceuticals, increasing the HRT from 1 to 15 days often results in a 15%–80% increase in removal efficiency, as it allows for the induction of specific catabolic enzymes regulated by QS [88]. Compared to traditional treatment, MBBR improves treatment performance through continuous movement of carrier media for enhancing the contact between biofilm and wastewater. Besides, this system has shown significant capability in removing micropollutants when combined with an aerobic process [89]. So, the integration of QS and biofilm dynamics transforms bioreactors from simple filters into synchronized metabolic engines capable of mineralizing complex medical pollutants. By optimizing physical parameters like HRT and counter‐diffusion, these systems maximize mass transfer and activate the specific catabolic enzymes needed to degrade recalcitrant pharmaceuticals.

Similarly, trickling filter systems also play a role in the treatment of wastewater from medical facilities. QS‐biofilm integration on filter media degrades pharmaceuticals and disinfectants, demonstrating the system’s effectiveness in treating complex wastewater streams. When the wastewater flows over biofilm, pollutants are degraded by communities of microbes [90]. The trickling filter at the secondary treatment stage acts as a critical aerobic zone where microorganisms in the biofilm metabolize dissolved medicinal residues. While it achieves nearly complete removal for ibuprofen (99%) and paracetamol (98%), it struggles more with higher concentrations of diclofenac, reaching only a 74% removal rate. Results indicate that the filter’s efficiency begins to decline when pharmaceutical concentrations, particularly paracetamol and diclofenac, exceed a threshold of roughly 80 μg/mL. To improve the final effluent quality, the assessment suggests supplementing the biological filter with advanced sorption or phototransformation techniques to capture the remaining recalcitrant compounds [91]. The advantage of trickling filters is known for their simplicity and cost‐effectiveness, making them suitable for various wastewater treatment applications [92]. A comprehensive summary of common biofilm bioreactors and their operational features is provided in Table 4.

**TABLE 4:** Biofilm bioreactors and their features.

Reactor type	Key feature	Advantages of pharmaceutical pollutants	Disadvantages/challenges	Reference
Membrane bioreactor (MBR)	Combines biological degradation with membrane filtration.	High‐quality effluent, low sludge production, effective for high organic concentrations and specific pollutants (e.g., ciprofloxacin, norfloxacin), stable process.	Membrane fouling is a significant challenge that can lead to increased maintenance costs.	[93]
Fluidized bed biofilm reactor (FBBR/FBR)	Uses small, fluidized carriers to increase surface area and mass transfer.	High removal efficiency (often > 90% for organics/ammonia), better temperature control than fixed beds, high biomass concentration.	High cost, potential for internal corrosion, requires significant energy for fluidization.	[94, 95]
Moving bed biofilm reactor (MBBR)	Uses free‐floating carriers for biomass attachment.	Space‐saving, low maintenance, high resistance to toxic environments and organic shock loads, and efficient in organic removal and nitrification/denitrification.	Carriers can be washed out of the system if sieves are not properly designed.	[81, 96]

##### 2.1.2.2. Electroactive Biofilms (EABs) and Microbial Fuel Cells (MFCs)

EABs are active microbial communities capable of extracellular electron transfer (EET) with electrodes and are used in bioremediation [97, 98]. These active microbial communities utilize direct and indirect mechanisms for electron transfer to and from electrodes [98]. The composition and structure of this active biofilm differ depending on whether they utilize the electrode as an electron acceptor or donor, with specific microbes enriched in each environment [99]. The major EAB‐forming species are *Geobacter*, *Dysgonomonas*, and various *Proteobacteria* [100]. Besides, EABs play a crucial role in pharmaceutical wastewater treatments [101]. These biofilms formed by electroactive microbes can directly exchange electrons with electrodes without any mediators [97]. This electron transfer efficiency in bio‐electrochemical systems is a crucial application for the decontamination of wastes [102]. Then, the effectiveness in detoxifying pharmaceutical wastes, maintaining their biochemical machinery intact despite harsh conditions [101].

In the bioremediation of EMOPs, EABs represent a transformative application where QS coordinates the dual processes of pharmaceutical degradation and EET [103]. In technologies such as MFCs, QS signaling molecules like AHLs and phenazines act as the regulatory command to synchronize the metabolic activity of electrogenic bacteria [104]. By integrating these signaling pathways, EABs can maintain a high metabolic flux, effectively utilizing pharmaceutical compounds as electron donors even under high‐toxicity conditions that would typically inhibit standard microbial processes [56].

Previous studies indicate that EAB facilitates the degradation of nonsteroidal anti‐inflammatory drugs like naproxen and ibuprofen through a series of processes [105]. Initially, the microbial community from wastewater attaches to the conductive anode surface and then forms a biofilm. Consequently, this biofilm uses those anodes as electron acceptors and enhances electron transfer during metabolic activities [106, 107]. After that, the degradation releases electrons that are transferred to the anode, generating a current that is known as an electrical current, and then further stimulating microbial mechanisms of activity [97]. Then the process forms an intermediate metabolite, which is metabolized by the biofilm, finally resulting in the complete degradation of the nonsteroidal anti‐inflammatory drugs into end products such as carbon dioxide, water, and biomass [108].

Moreover, specific case studies involving the treatment of sulfamethoxazole‐ and roxarsone‐contaminated wastewater demonstrate the power of this causal synergy [56]. Research has shown that the exogenous addition of the signaling molecule 3‐oxo‐C12‐HSL can increase the removal efficiency of persistent antibiotics by over 40% while simultaneously enhancing power density in MFC systems [109]. These results underscore that the synergy between QS and EABs provides a robust, self‐powering platform for the detoxification of recalcitrant medical waste, bridging the gap between waste treatment and sustainable energy recovery.

A MFC is an advanced mechanism for the degradation of medical wastes through the formation of biofilm [110]. In MFCs, on the anode surface, specific microbes form a biofilm, where they thrive on the organic substrate, including different pharmaceutical wastes [111, 112]. Firstly, when these microbes metabolize the toxic pharmaceutical wastes, they facilitate the transfer of electrons produced during their metabolic process to the anode [111]. During the degradation of bacteria, these wastes not only generate electrons but also protons, which are mitigated to the cathode through a proton exchange membrane. This electron transfer generates an electric current, effectively both energy production and environmental remediation [113].

Studies have explored the application of microbial fuel cells for the enhancement of biofilm and QS in the degradation of medical wastes such as diclofenac. Wu et al. [114] reported that the diclofenac removal rate after 2 weeks of microbial fuel cell operation was 30.73%. Likewise, Qiu et al. [115] described that degradation of diclofenac has been achieved using a modified anode, such as Ru/Fe‐modified electrodes, which improves the degradation performance. In addition, Morovati et al. [116] demonstrated that modifying MFC anodes with MnCo_2_O_4_ improved diclofenac removal efficiency to 56% after 48 h. These results underscore that the synergy between QS and microbial biofilms provides a robust, self‐powering platform for the detoxification of recalcitrant medical waste, bridging the gap in waste treatment.

##### 2.1.2.3. Genetic Engineering (GE) Technology

Conventional wastewater treatments are ineffective for removing emerging pollutants such as pharmaceuticals [117, 118]. To overcome these challenges, genetically modified microbes play a crucial role in enhancing the degradation of healthcare wastes through biofilm formation [119]. A genetically modified organism (GMO) is created by inserting proteins into bacteria through GE techniques, resulting in more potent organisms with greater degradative capacity to different pollutants [73].

In the context of bioremediation strategies, GE technology serves as the bridge between theoretical microbial signaling and practical, high‐efficiency removal of EMOPs [120]. By manipulating the genetic architecture of both the QS pathways and the biofilm‐forming genes, researchers can create “super‐degrader” strains tailored for specific pharmaceutical waste streams [121]. This involves the insertion of high‐affinity catabolic genes, such as those encoding for specialized dioxygenases or cytochrome P450 monooxygenases, into robust biofilm‐forming hosts [122]. The synergy is achieved by placing these degradation genes under the control of QS‐responsive promoters (e.g., *luxI/R* or *lasI/R*) [123], ensuring that the metabolic “machinery” for detoxifying complex drugs like CBZ or diclofenac is only activated when a stable, high‐density biofilm community is established, thereby preventing metabolic exhaustion of the microbes [124]. While GE offers a powerful pathway to enhance the degradation of recalcitrant pharmaceuticals, its application in open wastewater systems is governed by significant biosafety and regulatory constraints [121].

For instance, genetically modified Escherichia *coli* can develop the ability to degrade the antibiotic pollutant tetracycline in wastewater. Engineered *E. coli* can degrade toxic tetracycline in wastewater through biofilm formation [125]. Moreover, studies have successfully cloned and expressed a tetracycline‐degrading enzyme known as TetX from *Bacteroides fragilis* in *E. coli* and report the ability to degrade over 95% of tetracycline in 24 h [125, 126]. In this condition, a biofilm‐enhanced environment played a crucial role, which provided a stable habitat for the microbes by facilitating cell density and metabolic activities. Likewise, the biofilm protects bacteria from the potentially toxic effects of high antibiotic concentrations and then allows for degradation activities [31, 127]. This case study indicates the role of QS‐biofilm integration in degrading antibiotic residues by engineered *E. coli.*

Moreover, a significant breakthrough in this field involves the engineering of *Pseudomonas putida* strains modified to overexpress AHL synthases. In experimental trials, these genetically enhanced strains demonstrated a faster biofilm maturation rate and a significantly denser EPS matrix compared to wild‐type strains [128]. When applied to the treatment of hospital wastewater contaminated with sulfamethoxazole, these engineered biofilms maintained a removal efficiency of over 75% even during fluctuating influent concentrations [129]. This success is attributed to the “hard‐wired” synergy where the genetic modification ensures that signal production and metabolic degradation are intrinsically linked, providing a reliable and scalable solution for the specialized treatment of medical waste.

However, the transition from lab‐scale success to field application faces critical limitations and ecological risks. A primary concern is horizontal gene transfer (HGT); the dense microbial proximity within biofilms facilitates the accidental transfer of synthetic genetic constructs to indigenous environmental bacteria via conjugation or transformation [130]. If antibiotic‐resistance markers used in the engineering process escape into the wild, they could exacerbate the global crisis of multidrug resistance [131]. Furthermore, metabolic burden, where the energy required to maintain the synthetic pathway reduces the microbe’s overall fitness, often leads to the engineered strain being outcompeted by native populations in nonsterile wastewater environments [132].

From a regulatory perspective, the release of GMOs into municipal or hospital effluent is strictly prohibited in many jurisdictions due to the “escape” risk. To mitigate this, researchers are exploring biocontainment strategies, such as synthetic auxotrophy or “kill switches” that trigger cell death once the target pollutant is depleted [133]. Despite the high degradative potential of GE biofilms, their use remains restricted to contained bioreactor systems until the ecological consequences of gene flow and microbial displacement are fully understood and mitigated.

##### 2.1.2.4. Nanobioremediation

Nanotechnology acts as a transformative catalyst in the bioremediation of EMOPs by precision‐tuning the interplay between microbial communication and structural growth [134, 135]. By serving as high‐surface‐area scaffolds, nanomaterials enhance biofilm stability and promote the localized concentration of pollutants, increasing their bioavailability for microbial degradation [136]. Simultaneously, nanoparticles modulate QS pathways, either by delivering signal mimics that trigger the expression of degradative genes or by using “quorum quenching” techniques to prevent the formation of excessively thick, diffusion‐limited biofilms [137]. This synergy is further amplified through the use of functionalized nanomaterials that can generate reactive oxygen species to preoxidize complex pharmaceuticals or act as electron shuttles to accelerate microbial metabolism. Ultimately, nanotechnology transforms passive biofilms into highly efficient, engineered “living filters” capable of mineralizing resistant medical compounds that traditional treatment systems often fail to remove.

A significant application involves the use of chitosan‐coated silver nanoparticles to enhance the degradation of endocrine‐disrupting medical waste. In these systems, the nanoparticles serve as structural templates that accelerate biofilm formation by mimicking microbial attachment sites [138]. Research has shown that the integration of these nanoparticles into a QS‐active biofilm can increase the removal efficiency of persistent pharmaceuticals by a significant margin compared to traditional methods [135]. Furthermore, the conductive nature of certain nanomaterials has been found to facilitate EET within the biofilm, effectively speeding up the redox reactions required to mineralize complex medical compounds [139]. This integrated nano‐bio approach, detailed in the latest nano reports, provides a modular and resilient platform for addressing the most recalcitrant medical pollutants in water systems.

##### 2.1.2.5. Co‐Culture Systems

Co‐culture systems have an excellent strategy for degrading healthcare pollutants by combining the metabolic capabilities of various microbial species [140]. These systems break down complex compounds by cooperating with the metabolic pathway, where one species of metabolite can be further broken down by another [141]. This system has shown promise in degrading healthcare wastes with reduced accumulation of toxic intermediates [140]. Furthermore, co‐culture systems such as fungal‐bacterial consortia can achieve higher degradation rates for diclofenac, CBZ, and ketoprofen [140]. These co‐cultures benefit from mechanisms such as co‐degradation and enhanced enzymatic synthesis [142].

Moreover, co‐culture systems represent the pinnacle of microbial communities, leveraging the causal synergy between QS and biofilm dynamics to tackle complex medical organic pollutants that exceed the metabolic capacity of single strains [143]. Unlike monocultures, these systems employ a “division of labor” strategy in which diverse species—such as specialized degraders and robust biofilm producers—are synchronized through interspecies signaling molecules, such as Autoinducer‐2 (AI‐2) [144]. This signaling ensures that the physical protection of the biofilm (provided by one species) is perfectly timed with the metabolic activation of catabolic enzymes (in another species), creating a stable microenvironment for the sequential breakdown of multidrug cocktails.

Studies have shown that CBZ was metabolized through a mixed culture of *Leifsonia shinshuensis* and *Rhodococcus zopfii* using oxygen oxidoreductase and aldehyde oxidase enzymes. Initially, the breakdown of CBZ is initiated by *L. shinshuensis,* then leads to the complete mineralization of the resulting metabolites through *R. zopfii* [145]. The close relationship of the two mixed species within the biofilm enhanced the exchange of nutrients and more metabolic cooperation [146]. This case study shows that the combination of mixed cultures can enhance QS and biofilm and improve the degradation of CBZ, thereby offering an effective method for environmental remediation.

### 2.2. Case Studies of QS and Biofilm Synergistically Degrade EMOPs

The integration of QS and biofilm synergistically in medical EOP degradation follows the principle of coordinated metabolic labor‐sharing, where signal molecules synchronize the kinetics of complex pollutant breakdown. QS‐mediated approaches have demonstrated a transition from first‐order to zero‐order kinetics as microbial density increases [60]. For example, Cheng et al. [103] described that QS signal molecules enhance chlortetracycline degradation by up to 56.53% in MFCs. While *Serratia marcescens* strain WW1 achieved a degradation rate constant (*k*) that allowed for 89.5% removal within 48 h [147]. The findings from recent research highlight the causal synergy between QS and biofilm dynamics by showing that signaling molecules do not just build the “house” (biofilm) but also activate the specific “machinery” inside it to degrade medical pollutants.

**FIGURE 1:**
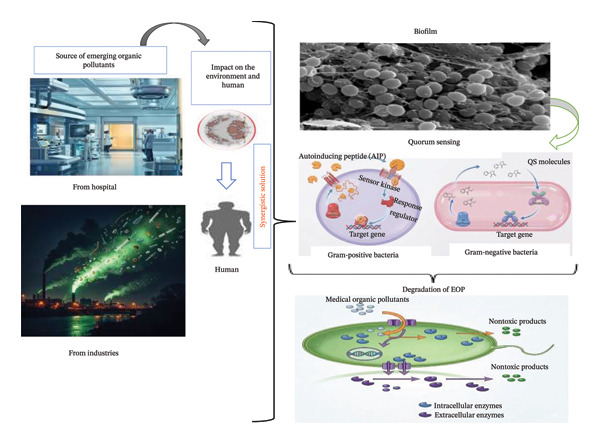
Synergies of quorum sensing and biofilm dynamics in the degradation of emerging medical organic pollutants.

Generalizable principles of microbial cooperation are further evidenced in the treatment of antidepressants [140, 148]. Additionally, work by Duarte et al. [149] and Fernandes et al. [148] demonstrates that the use of bacterial consortia, dominated by *Pseudomonas* and *Acinetobacter*, achieves superior removal, over 97% for paroxetine and bezafibrate, compared to monocultures. This efficacy is attributed to the synergistic degradation of metabolites; one species may perform the initial hydroxylation, while another mineralizes the resulting intermediate, thereby reducing overall effluent toxicity. A similar study by Palma & Costa [54] reported the anaerobic biodegradation of fluoxetine by a bacterial community containing sulfate‐reducing bacteria. This approach indicates the potential effectiveness of QS and biofilm in the bioremediation of pharmaceutical waste using microbial cooperation, as shown in Figure 1.

## 3. Conclusion

**FIGURE 2:**
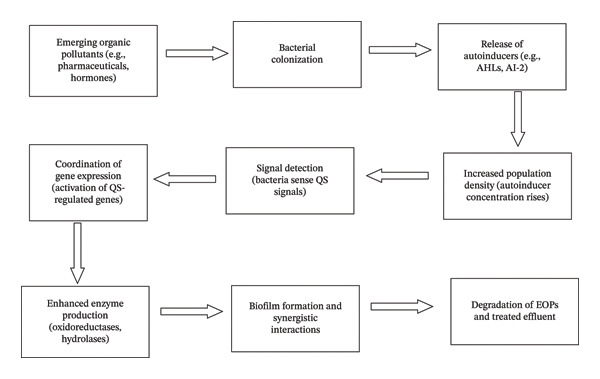
Summary diagrammatic representation of biofilm and QS in the degradation of emerging medical organic pollutants (constructed by author).

Generally, this review demonstrates that the synergy between biofilm dynamics and QS provides a robust framework for the removal of EMOPs, as shown in Figure 2. Critical analysis reveals that biofilms offer a protected microenvironment for specialized metabolic pathways, while QS functions as a regulatory essential for synchronizing the secretion of catabolic enzymes like laccases and hydrolases. Likewise, it also emphasizes the significance of QS in coordinating bacterial activities, activating specific enzymes that play a role in degradation, and promoting synergistic interactions within biofilm communities. Moreover, the case studies presented prove the effectiveness of these strategies in the treatment of wastewater contaminated with EOPs, such as antibiotics and antidepressants. However, several critical gaps remain: First, the transition from lab‐scale case studies to full‐scale bioreactors is often hindered by the “metabolic burden” on engineered strains and the ecological instability of microbial consortia under fluctuating hospital influent loads. Second, while high removal efficiencies (up to 97%) are achievable for parent compounds like antidepressants, the potential for accumulating toxic metabolites requires more rigorous monitoring of effluent toxicity rather than just concentration reduction. Therefore, future research must prioritize the development of robust biocontainment strategies for GMOs and the optimization of hydraulic retention times to ensure complete mineralization. Rather than serving as a simple basis for action, this review provides a strategic roadmap for integrating QS‐mediated regulation into existing wastewater infrastructure to achieve the sustainable and safe remediation of healthcare‐derived EOPs. Overall, the paper underscores the application of both advanced biotechnological strategies and wastewater treatment processes to develop sustainable solutions for healthcare EOPs.

## 4. Challenges and Future Directions

A critical analysis of integrated QS and biofilm systems reveals several significant limitations that hinder real‐world application. A primary challenge involves regulatory and public acceptance hurdles concerning the environmental release of genetically modified microorganisms (GMMs) designed for enhanced degradation, necessitating complex and costly biocontainment strategies. For EABs used in MFCs, the transition from lab to pilot scale is hampered by prohibitive costs associated with specialized electrode materials and persistent performance inconsistencies in large systems. Furthermore, while mixed microbial cultures are promising, maintaining long‐term community stability and optimal species composition in highly variable wastewater matrices is challenging. The efficacy of QS‐regulated systems is also threatened by environmental signal instability (e.g., pH and temperature) and interference from indigenous quorum‐quenching microorganisms, which can neutralize signaling molecules. Ultimately, these biological and technical complexities create a gap between laboratory success and the consistent, cost‐effective performance required for full‐scale commercial deployment.

Therefore, for future research, further exploration of advanced GE techniques is required to develop more efficient engineered microbes for wider degradation of EOPs that are found in healthcare waste. This could involve enhancing enzymatic activities, optimizing metabolic pathways, and improving adaptability to harsh environmental conditions. Furthermore, advancing the application of EAB in bioelectrical systems for both the treatment of healthcare waste and the generation of renewable energy is crucial. Moreover, investigating the synergistic interactions between different microbial species in mixed culture systems for enhanced biodegradation of complex medical waste is critical. Optimizing the composition of these mixed cultures could lead to efficient degradation strategies.

Besides, specific key research in QS and biofilm‐regulated remediation technology must also be a future direction. Including (i) research on developing precise biofilm control strategies using QS manipulation and optimizing the architecture and function of biofilm for targeted pollutant degradation. (ii) Investigating the stability of QS signals in the degradation of the wastewater pathway, which can be influenced by several environmental factors such as temperature, pH, and indigenous quorum‐quenching microbes. Finally, real‐scale validation and pilot‐scale deployment of QS‐biofilm–enhanced bioremediation systems are essential steps to transition this promising lab scale to a practical way.

## Author Contributions

Yohannes Tsegay Teklay contributed to the conception and design, literature search and analysis, drafted the manuscript, and made critical revisions.

## Funding

This review did not receive any specific grant from funding agencies in the public, commercial, or not‐for‐profit sectors.

## Disclosure

Yohannes Tsegay Teklay approved the final version of the review.

## Conflicts of Interest

The author declares no conflicts of interest.

## Data Availability

All the datasets generated in this review are included within the article.
